# The Life Cycle of *L. pneumophila*: Cellular Differentiation Is Linked to Virulence and Metabolism

**DOI:** 10.3389/fcimb.2018.00003

**Published:** 2018-01-19

**Authors:** Giulia Oliva, Tobias Sahr, Carmen Buchrieser

**Affiliations:** ^1^Institut Pasteur, Biologie des Bactéries Intracellulaires, Paris, France; ^2^Centre National de la Recherche Scientifique, UMR 3525, Paris, France

**Keywords:** *Legionella pneumophila*, regulation, virulence, metabolism, life cycle

## Abstract

*Legionella pneumophila* is a gram-negative bacterium that inhabits freshwater ecosystems, where it is present in biofilm or as planktonic form. *L. pneumophila* is mainly found associated with protozoa, which serve as protection from hostile environments and as replication niche. If inhaled within aerosols, *L. pneumophila* is also able to infect and replicate in human alveolar macrophages, eventually causing the Legionnaires' disease. The transition between intracellular and extracellular environments triggers a differentiation program in which metabolic as well as morphogenetic changes occur. We here describe the current knowledge on how the different developmental states of this bacterium are regulated, with a particular emphasis on the stringent response activated during the transition from the replicative phase to the infectious phase and the metabolic features going in hand. We propose that the cellular differentiation of this intracellular pathogen is closely associated to key metabolic changes in the bacterium and the host cell, which together have a crucial role in the regulation of *L. pneumophila* virulence.

## Environmental and morphogenetic changes

Whatever strategy microbial pathogens have evolved to successfully infect and replicate in their hosts, they have adapted in the course of evolution to face hostile environments. This adaptation allows them to benefit from the environment of the susceptible host cell and simultaneously to ensure their persistence for another infection cycle. A conspicuous group of bacteria, referred to as facultative and obligate intracellular pathogens, exploits a variety of different hosts to establish a cytosolic or vacuolar niche for replication. Thereby they face and learned to tolerate acidification, starvation, and changes in temperature, oxidative stress and many other host defense mechanisms. Most of these bacteria are also located in the extracellular space between intracellular infection cycles and display thus a dual intracellular/extracellular lifestyle. Among those, the intracellular pathogen *Legionella pneumophila* thrives in fresh water environments, where it either spreads planktonically as free-living microbe or it is associated with biofilm communities (Steinert et al., 2002; Hilbi et al., 2011), but it never has been demonstrated to replicate in these environments. In the environment *Legionella* replicate within eukaryotic phagocytic cells like the environmental amoeba *Acanthamoeba castellanii*, as well as in human monocytes and alveolar macrophages (Horwitz and Silverstein, 1980; Rowbotham, 1986). *L. pneumophila* has successfully adapted to new and challenging environments created by human activities, such as showers, air conditioning systems, water fountains, cooling towers or other artificial water systems facilitating access to humans and human infection, which can result in a severe pneumonia, called Legionnaire's disease or legionellosis (McDade et al., 1977). However, mainly the susceptible population like immunocompromised or elderly persons develop pneumonia caused by *Legionella*, as this bacterium evolved with aquatic protozoa and thus it has not evolved mechanisms to counteract the host defense in healthy humans. In addition to biofilm communities and protozoan predators, *L. pneumophila* has been found to colonize more extreme environmental niches, such as antarctic freshwater lakes at temperature at 0°C as well as extremely acidic habitats and water sources with temperature over 60°C (Hilbi et al., 2011). Therefore, *L. pneumophila* endures in disparate environmental conditions throughout its life cycle with respect to nutrient access and availability, pH, temperature, and host defenses during intracellular replication. The transition between intracellular and extracellular habitats triggers morphogenetic and metabolic changes during the bacterial lifecycle (Molofsky and Swanson, 2004). Accordingly, *L. pneumophila* alternates between different morphogenetic forms including a replicating form (RF), and a transmissive/virulent form that have many distinct features (Molofsky and Swanson, 2004; Brüggemann et al., 2006; Steinert et al., 2007). Starvation and environmental stress induce the transition from the metabolically active, replicating bacteria to motile, stress-resistant virulent bacteria (Molofsky and Swanson, 2004). Moreover, a mature intracellular form (MIF), characterized by bacteria that are highly infectious, motile and cyst-like was described (Garduño et al., 2002; Robertson et al., 2014) as well as viable but non-cultivable (VBNC) forms that develop in response to disparate conditions (Steinert et al., 1997; Al-Bana et al., 2014). The fine-tuned regulation of these different forms ensures the persistence and successful life cycle of this bacterium. Thus, *L. pneumophila* employs a multitude of regulatory elements allowing it to govern its multi-phasic life cycle.

## One strategy, multiple hosts

In the environment, *L. pneumophila* preferentially establishes a parasitic relationship with protozoa, which provides not only a nutrition source for the persistence, replication and dissemination of *Legionella*, but also functions as shelter offering protection from adverse environmental conditions (Barker et al., 1995; Cunha et al., 2016). Interestingly, bacteria released from protozoa are more infectious, are highly motile and more efficient in surviving and multiplying within human monocytes *in vitro* compared to bacteria grown on agar (Cirillo et al., 1994; Brieland et al., 1997). The protozoan predators (amoebae and ciliates) are the natural hosts of *L. pneumophila*, and humans are accidental hosts as judged by the evidence that only a single and recent case of human-to-human transmission has been reported to date (Correia et al., 2016). Thus, *L. pneumophila* transmission to humans occurs primarily from man-made environmental sources (Hilbi et al., 2010; Newton et al., 2010). The dual host specificity of *Legionella* is thought to be derived from the fact that protozoa are primordial phagocytes and as such they share many similarity at both cellular and molecular level with macrophages. Therefore, the intracellular growth of *L. pneumophila* is very similar in both hosts (Fields et al., 2002; Hilbi et al., 2007) suggesting that the co-evolution of *Legionella* within protozoa had provided the bacterium with an effective strategy to colonize two evolutionally different host cells. Indeed, this co-evolution is reflected in its genome as sequence analyses revealed that *L. pneumophila* as well as *L. longbeachae* have acquired genes coding for proteins with eukaryotic-like properties from its protozoan predators (Cazalet et al., 2004, 2010; de Felipe et al., 2005; Gomez-Valero et al., 2011). These eukaryotic-like proteins were shown to be secreted effectors that mimic the functions of their host counterparts (Cazalet et al., 2004; de Felipe et al., 2005; Nora et al., 2009; Gomez-Valero et al., 2011; Escoll et al., 2016). Their translocation to the host cell is achieved by the Dot/Icm type 4B secretion system (T4BSS), which is indispensible for intracellular replication of this bacterium (Ninio and Roy, 2007; Isberg et al., 2009; Zhu et al., 2011). Thus, this intriguing feature of molecular mimicry is a major virulence strategy developed by this opportunistic bacterium due to a selective pressure from the natural environment (Nora et al., 2009; Escoll et al., 2016).

## Human infection

Adaptation of *L. pneumophila* to harsh environmental conditions allowed them to become ubiquitous in human-made aquatic systems where the temperature is higher than the ambient temperature. As consequence, thermally altered aquatic habitats may shift the availability of predators and bacterial preys, eventually promoting *Legionella* replication and the emergence of the disease by inhalation of infected aerosols. Potential sources of *Legionella* transmission include potable water sources, such as fountains, showers and taps, and non-potable sources such as spas, cooling towers and evaporative condensers (Steinert et al., 2007; Newton et al., 2010; Cunha et al., 2016). Upon inhalation of bacteria-contaminated aerosols, *Legionella* reach the lung and are engulfed by alveolar macrophages wherein they can actively replicate, causing a life-threatening pneumonia called Legionnaires' disease (Newton et al., 2010). As *Legionella* is an opportunistic pathogen, persons with chronic lung diseases, elderly, immune-compromised, male gender and smokers are mainly susceptible to contract the disease (Newton et al., 2010; Cunha et al., 2016). Interestingly, not all *Legionella* species seem to be able to cause human disease as among the 58 *Legionella* species currently identified, only about 20 have been associated to human disease. Among those, *L. pneumophila* serogroup 1 is responsible for over 85% of the Legionnaires' disease cases world-wide (Yu et al., 2002; Newton et al., 2010)

## Life within A host cell

**Figure 1:**
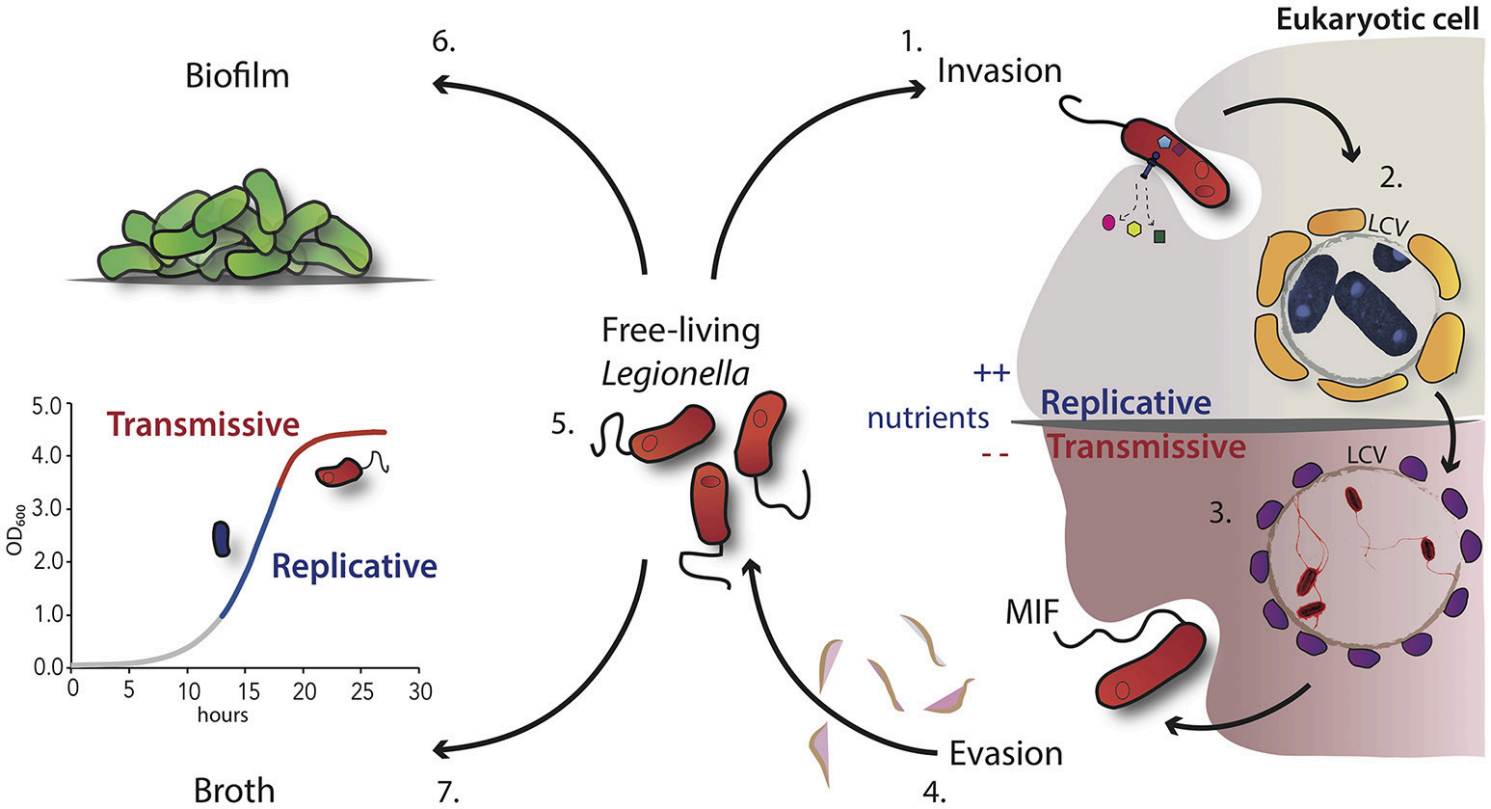
Schematic overview of the *L. pneumophila* morphological states during its growth cycle. 1. Uptake of virulent *L. pneumophila* by the host cell like protozoa or macrophages through convention or coiling phagocytosis. 2. After internalization, the bacteria evade the phagosome-lysosome fusion and start the intracellular multiplication within the LCV, which is surrounded by vesicles (in yellow) rich in lipids and proteins. 3. Nutrient starvation induces the activation of the stringent response and morphological changes. Bacteria express the transmissive traits such as motility (flagella) and become cytotoxic. 4. These infectious bacteria are able to lyse the vacuolar membrane and are released in the extracellular environment. 5. Free-living transmissive bacteria may start a new cycle or persist in the extracellular environment as planktonic form. 6. Alternatively, *L. pneumophila* may be associated within biofilms, either in natural fresh-water habitats or artificial ones. 7. In broth culture, *L. pneumophila* displays also a biphasic life cycle, which closely mimics the replicative and transmissive intracellular forms.

One of the striking features of *L. pneumophila* is its ability to replicate within a large number of different host cells. The intracellular lifestyle and the adaptation capacity require a series of temporally distinct events leading to the establishment of a successful infection cycle, many of which are provoked by the action of one or more of the over 300 effector proteins known to be secreted by the Dot/Icm secretion system. Following the uptake of *L. pneumophila* by phagocytic cells through conventional or coiling phagocytosis (Bozue and Johnson, 1996; Escoll et al., 2013), this bacterium avoids lysosome-mediated degradation and forms a unique replication-permissive compartment within its host cell (Figure 1). This single-membrane *Legionella* containing vacuole (LCV) differs from the cellular compartment containing non-pathogenic bacteria since it does not acidify and it has a distinct membrane identity that is achieved by the recruitment of vesicles rich in lipids and proteins on the cytoplasmic face. Four hours after entry into phagocytes, vesicles derived from rough endoplasmic reticulum (ER) cluster near the nascent LCV. Based on the localization of ER-associated proteins within the LCV, these vesicles, which exit the ER, are able to deliver their content into the vacuoles containing *L. pneumophila* (Robinson and Roy, 2006). In this compartment the bacteria are replicating intravacuolarly, but the LCV was found later in association with the late lysosomal compartment, suggesting that it may also play a role in the replication of the bacteria by providing a nutrient-rich environment (Sturgill-Koszycki and Swanson, 2000). Recent studies demonstrated that *L. pneumophila* evades the host-cell response and interferes also with the host autophagy machinery by modulating the host sphingolipid metabolism or autophagosome formation (Choy et al., 2012; Rolando et al., 2016). The question remains open, whether the manipulation of the host sphingolipd metabolism may not only modulate autophagy, but also provide *L. pneumophila* nutrients for replication. However, additional membrane trafficking events may occur and modulate the intracellular life cycle of this bacterium to manipulate the host response. Following intracellular multiplication, the depletion of nutrients triggers morphological changes and a switch from a replicative form, where bacteria are metabolically active but not infectious, to a transmissive form, which ensures that the bacteria activate the infectious traits for the escape and transmission into a new suitable host or the survival in the environment (Byrne and Swanson, 1998; Garduño et al., 2002; Molofsky and Swanson, 2004; Robertson et al., 2014; Figure 1).

## The biphasic life cycle of *L. pneumophila*

### *L. pneumophila* undergoes morphologic changes during its life cycle

In a simple model one can describe the *L. pneumophila* life cycle as alternating between two distinct and reciprocal forms: a replicative and a transmissive form that was referred to as microbial differentiation (Molofsky and Swanson, 2004). This term implies physiological, morphogenetic and metabolic changes of the bacterium. Indeed, pronounced morphogenetic changes in the bacterial cell wall, the bacterial shape and in motility as well as the enrichment of the cell in energy-rich polymers are observed (Rowbotham, 1986). Within the LCV, acid-resistant, replicating bacteria exploit the nutrient-rich environment and actively inhibit the phagosome-lysosome fusion to be able to efficiently multiply. Therefore, bacterial density strongly increases whereas nutrient access dramatically decreases over time. Actively replicating bacteria appear as slender rods, are non-motile and display a wavy cell wall (Faulkner and Garduño, 2002). During this metabolically active state, traits related to virulence and transmission such as motility and cytotoxicity are not required thus the replicating bacteria either lack an activator of transmission and/or constitutively express a repressor of transmission traits (Byrne and Swanson, 1998; Molofsky and Swanson, 2003). As local nutrient levels become limiting and disadvantageous conditions are about to be faced, the bacteria convert into the infectious/transmissive variant. Interestingly, virulent bacteria appear as short, stubby rods with blunt ends containing cyst-like inclusions of poly-3-hydroxybutyrate (PHB), and display a smooth thick cell wall (Faulkner and Garduño, 2002). Those phenotypically distinct bacteria coordinately activate the expression of the so-called transmissive traits, which are required for lysosome evasion, escape from the spent host cell, survival in the extracellular environment and the invasion of a new suitable host. After successfully establishing a new intracellular niche, *L. pneumophila* reverts to its replicative form, starting a new cycle (Hammer and Swanson, 1999; Molofsky and Swanson, 2004). To limit costly energy levels, *L. pneumophila* has adopted a strategy employing a reciprocal biphasic expression pathway. Therefore, when conditions are favorable for multiplication, the virulence traits are neither required nor expressed. Conversely, in adverse conditions such as the nutrient deprivation, the bacteria do not replicate (Byrne and Swanson, 1998). Strikingly, the analyses of the global gene expression profiles of *L. pneumophila* in the *in vivo* infection model *A. castellanii* as well as in the *in vitro* broth culture model revealed that the pathogen's life cycle is very similar, as judged by the profound and similar changes in the gene expression program from the replicative/exponential growth phase to the transmissive/post-exponential growth phase of the bacteria (Brüggemann et al., 2006; Faucher et al., 2011). Thus, replicative and transmissive bacteria share a gene expression program similar to that of *in vitro* grown exponential (E) and post-exponential (PE) bacteria, respectively, suggesting that the biphasic life cycle is globally controlled by the bacterial growth phase and by nutrient availability. In addition, intracellular infection of the natural host *A. castellanii* revealed only few strain-specific differences, such as a shorter lag phase of strain *L. pneumophila* Paris and an earlier transition to transmissive form (Brüggemann et al., 2006). Interestingly, the global expression profiles of replicative and transmissive phases of three different *L. pneumophila* strains have been shown to be very similar (Brüggemann et al., 2006). In contrast, comparison of the gene expression program of the E and PE phases of the two major disease-associated species *L. pneumophila* and *L. longbeachae*, revealed clear differences. Particularly, the transition between the replicative and transmissive form is less pronounced in *L. longbeachae*, which seems to regulate this transition mainly by engaging secondary messenger molecules and less transcriptional and post- transcriptional regulators than *L. pneumophila* (Cazalet et al., 2010). Taken together, the transition from the exponential/replicative to the post-exponential/transmissive phase governs a common virulence program engaged of *L. pneumophila* within host cells (Brüggemann et al., 2006; Faucher et al., 2011).

### The key metabolic capacity of *L. pneumophila* is adapted to its biphasic life cycle

In response to fluctuating intracellular environmental conditions, *L. pneumophila* certainly requires a well-balanced adaptation of its metabolism. Main questions are (i) what are the essential nutrients required for intracellular proliferation of *L. pneumophila* during infection, (ii) what is the nutrient availability in the LCV and (iii) what are the capacities of the bacterium to catabolize these compounds. The development of a chemical defined liquid medium gave first insights into the nature and physiology of this intracellular pathogen by suggesting that it utilizes only amino acids as energy and carbon sources (Pine et al., 1979; George et al., 1980; Ristroph et al., 1981; Tesh et al., 1983). While formulating this medium, it has been demonstrated that cysteine was an absolute requirement for the bacterial growth and that the addition of soluble ferric pyrophosphate had stimulatory effects. Unlike other microorganisms, *L. pneumophila* has been found to use serine and threonine as a primary supply for energy production rather than any other organic substrate (Pine et al., 1979; George et al., 1980; Ristroph et al., 1981; Fields, 1996). Accordingly, microarray analyses performed during replicative growth of *L. pneumophila* either in broth or upon infection of *A. castellanii*, revealed that while replicating, bacteria express genes indicating that an aerobic metabolism and amino acid catabolism, particularly for serine, threonine, glycine, tyrosine, alanine, and histidine is taking place (Sauer et al., 2005; Brüggemann et al., 2006). However, unexpectedly the up-regulation of genes encoding the Entner-Doudoroff (ED) pathway, as well as of a putative glucokinase, a sugar transporter and the myo-inositol catabolism indicated that *L. pneumophila* may be also able to exploit host carbohydrate-derivatives during the replicative phase of growth within amoebae (Brüggemann et al., 2006). Interestingly, these analyses suggested for the first time that intracellular *L. pneumophila* also may utilize starch or glycogen as judged by the expression of a eukaryotic-like glucoamylase (GamA) during exponential growth (Brüggemann et al., 2006). Indeed, later, it was shown that GamA is responsible for glycogen- and starch-degrading activities of *L. pneumophila* and that it is expressed and active during intracellular replication in *A. castellanii*, suggesting that *L. pneumophila* is degrading glycogen during intracellular replication (Herrmann et al., 2011). Hence, intracellular *L. pneumophila* not only uses amino acids but also diverse carbohydrates as carbon supply (Weiss et al., 1980; Eylert et al., 2010).

**Figure 2:**
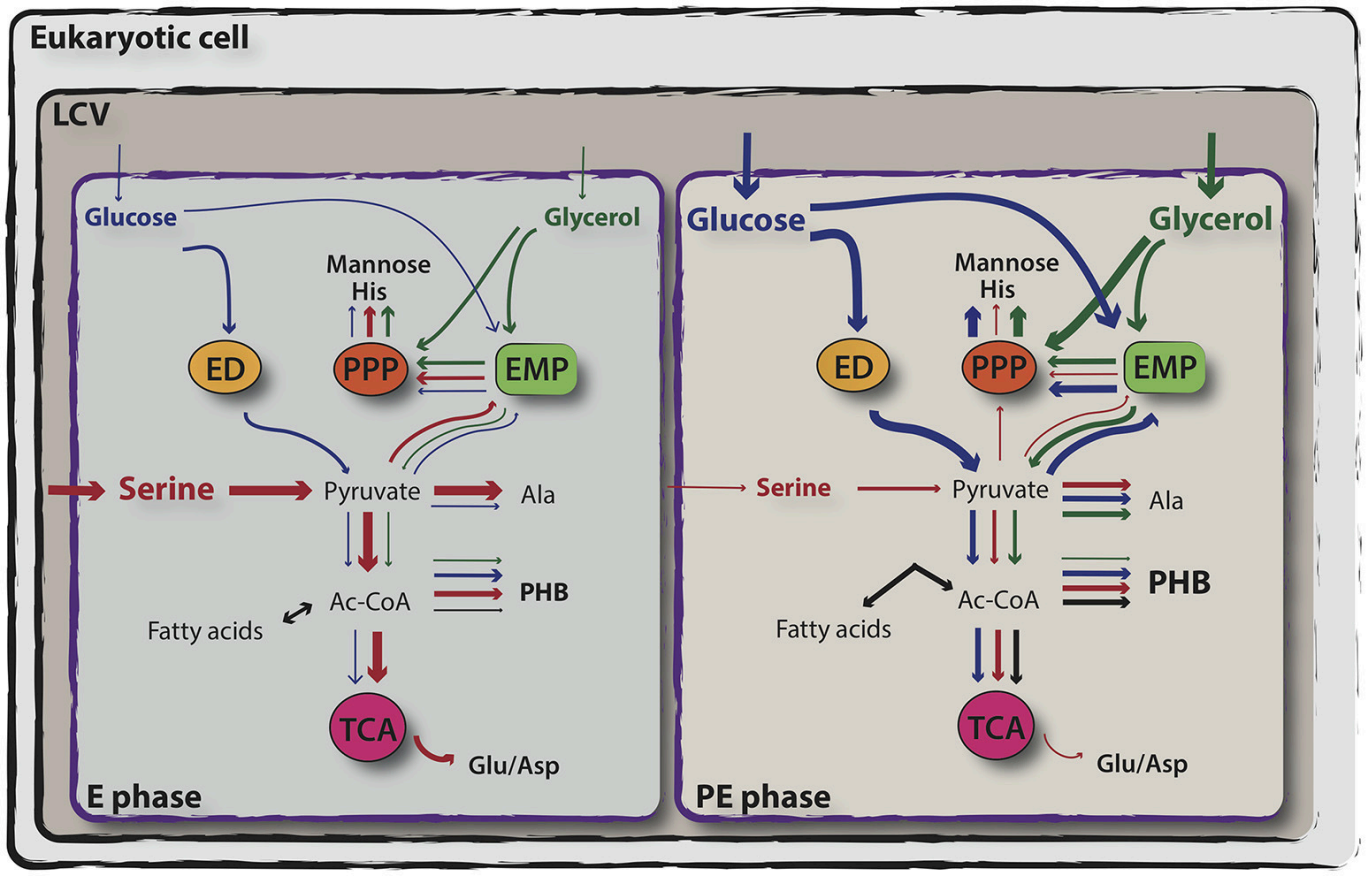
Simplified representation of exponential and post-exponential phase-dependent utilization of serine, glucose and glycerol by *L. pneumophila*. *In vitro* isotopologue labeling experiments using ^13^C-serine, ^13^C-glucose and ^13^C-glycerol revealed a bipartite metabolism in which serine (in red) is mainly used during the exponential phase of growth and enters primarily the TCA cycle, whereas glucose (in blue) and glycerol (in green) are shuffled into anabolic processes during the post-exponential growth phase of *L. pneumophila*. Relative carbon fluxes are depicted by the thickness of the arrows. For more detail, see the text. ED, Entner–Doudoroff pathway; PPP, pentose phosphate pathway; EMP, Embden-Meyerhof-Parnas pathway (glycolysis); TCA, tricarboxylic acid cycle (adapted from Eisenreich and Heuner, 2016).

However, *L. pneumophila* is auxotrophic for the amino acids Arg, Cys, Ile, Leu, Met, Thr, Val, Ser, Pro, and Phe (Pine et al., 1979; George et al., 1980; Ristroph et al., 1981; Tesh et al., 1983). Moreover, ^13^C- isotopologue profiling revealed that *L. pneumophila* is able to perform gluconeogenesis and to use the pentose phosphate pathway (PPP), although not all the genes encoding canonical enzymes involved in these pathways are present (Eylert et al., 2010). Based on the presence of a glucose transporter protein and on ^13^C-tracer experiments, it was reported that glucose, metabolized through the ED and PPP pathways serves as co-substrate for *L. pneumophila*, although the addition of glucose in broth culture does not increase the bacterial growth rate (Tesh et al., 1983; Eylert et al., 2010; Eisenreich and Heuner, 2016; Häuslein et al., 2016). Isotopologue profiling of key metabolites of *L. pneumophila* unveiled a bi-partite metabolism, in which it preferentially uses serine as major carbon, nitrogen and energy supply, whereas glycerol and glucose are shuffled into anabolic processes (Eisenreich and Heuner, 2016; Häuslein et al., 2016; Figure 2). In addition, as expected from an intracellular bacterium, which engages a growth phase-dependent program to control its virulence, it was shown that also the carbon and energy sources are metabolized in dependence of the growth phase (Gillmaier et al., 2016; Häuslein et al., 2016). As such, Serine is mainly metabolized during the replicative phase for amino acid (Ser> Ala >Glu> Asp = Gly) and protein biosynthesis (>50 mol%) and for energy production. ^13^C-labeled serine was found to enter mainly the TCA cycle, generating pyruvate and then acetyl-CoA, and to produce PHB (Eylert et al., 2010; Gillmaier et al., 2016; Häuslein et al., 2016). Conversely, during the post-exponential phase, despite the availability of serine in the medium, serine-dependent protein biosynthesis appears to be reduced, whereas carbon from serine is still used for PHB and fatty acid biosynthesis until the post-exponential phase of growth. Hence, upon entry into the stationary phase and under nutrient starvation, the PHB produced is catabolized by *L. pneumophila*, serving as main carbon and energy storage (James et al., 1999; Eylert et al., 2010; Gillmaier et al., 2016; Häuslein et al., 2016; Figure 2).

The other player in this bi-partite metabolism is glucose, which in *L. pneumophila* predominantly enters into the PPP for *the novo* production of histidine and sugars (in particular mannose) and that is also used in lower amounts for the synthesis of other amino acids and PHB. Conversely to serine, glucose is mainly metabolized throughout the late exponential and post-exponential phase of growth (Figure 2). As previously mentioned, *L. pneumophila* uses mainly the ED pathway, the gluconeogenesis and the PPP, and to a minor extent the glycolysis to metabolize glucose (Harada et al., 2010; Häuslein et al., 2016). Furthermore, glucose metabolism through the ED pathway is necessary for full fitness of *L. pneumophila* during its biphasic life cycle (Eylert et al., 2010). Earlier studies provided the first indication that glycerol may be used by *L. pneumophila* as carbon source, as deduced from the upregulation of a glycerol-3-phospate dehydrogenase (*glpD*) during intracellular growth in human macrophages (Faucher et al., 2011). Indeed, glycerol is predominantly metabolized during the late and post-exponential phase of growth like the life stage dependent usage of other carbon sources. Similar to glucose, the carbon from glycerol is mainly shuffled into gluconeogenesis and PPP for histidine and mannose production, but only low flux rates of carbon from glycerol into the TCA cycle were reported (Häuslein et al., 2016). In contrast, saturated lipids like palmitate, another carbon source shown to be predominantly used after amino acid depletion, is used for energy production and PHB synthesis (Häuslein et al., 2017). Taken together, the results from ^13^C- isotopologue profiling and flux analyses suggested that the life cycle switch of *Legionella* is also reflected by a metabolic shift from amino acids usage during replication to glycerolipids and glucose when entering transmissive phase (Häuslein et al., 2017; Figure 2).

### The metabolism of intracellular *L. pneumophila*

The acquisition of nutrients within host cells is an indispensable prerequisite for *L. pneumophila* multiplication and for a successful infection cycle. The presence and the up-regulation of genes encoding 12 different ABC transporters, amino acid permeases, proteases and phospholipases during intracellular multiplication within host cells suggested how *L. pneumophila* exploits host nutrient to support its intracellular growth (Brüggemann et al., 2006). Indeed, it was shown that intracellular replication of *L. pneumophila* depends on the host cell amino acid transporter SLC1A5 (Wieland et al., 2005) and that it employs the phagosomal transporter A (PhtA) to acquire threonine during growth (Sauer et al., 2005). Furthermore, ^13^C-Isotopologue compositions of amino acids from bacterial and amoebal proteins showed that *L. pneumophila* takes indeed amino acids up from its host (Schunder et al., 2014). In addition to the above mentioned transport systems, *L. pneumophila* was also reported to employ its effector proteins to generate nutrients for its growth. The *L. pneumophila* effector AnkB (Price et al., 2009; Lomma et al., 2010) is secreted in the host cell where it poly-ubiquitinates its targets leading to their proteolysis by the host proteasome. Price and colleagues suggested that in this way AnkB may generate short peptides and amino acids, which represent nutrients for intracellular bacterial multiplication as these free amino acids may be imported into the LCV *via* different host solute carriers and transporters, such as the glucose (Slc2a1, Slc2a6) and glycerol transporters (Slc37a1) (Price et al., 2011). A recent study reported opposing effects of two Dot/Icm secreted effector families, Lgt and SidE on the master regulator of host amino acid metabolism, the mechanistic target of rapamycin complex 1 (mTORC1). However, these two-effector families work synergistically to inhibit host translation and thereby liberate amino acids for *L. pneumophila* growth (De Leon et al., 2017). Thus, *L. pneumophila* not only employs its own transport systems for the uptake and to use amino acids but also seem to exploit the host proteasome machinery and mTORC1 to generate nutrients. Isoptopologue profiling during the replicative intracellular growth phase showed that *L. pneumophila* uses serine and other amino acids as main carbon and energy sources for protein biosynthesis, amino acid biosynthesis and PHB production (Price et al., 2011). Although less is known about the intracellular metabolism in the late phase of growth, it is likely that *L. pneumophila* gets access to carbohydrate sources such as glycogen, glucose and other polymers from the host upon LCV lysis (Lang and Flieger, 2011). In addition, the transmissive form of *L. pneumophila* contains high amounts of PHB, which serves as energy and carbon storage for the maintenance of the intracellular growth cycle (Gillmaier et al., 2016; Häuslein et al., 2017). Moreover, intracellular *L. pneumophila* metabolizes *myo*-inositol, which was reported to promote infection of *A. castellanii* and macrophages (Manske et al., 2016), and engages the translocated protein MavN, which once integrated in the host-derived LCV membrane, facilitates the acquisition of iron into its vacuole (Isaac et al., 2015). Once nutrients are limited in the LCV, this may be the signal for *L. pneumophila* to activate the infectious traits to escape the spent host. This transition from the replicative to the transmissive/virulent phase is highly regulated by a complex regulatory network, described in the following sections.

### Regulation of *L. pneumophila* differentiation

#### Triggers of the stringent response

After replicating within the LCV to high numbers, nutrients become limited, which triggers complex and coordinated regulation to allow the expression of transmissive traits, which provide *L. pneumophila* with the ability of leaving the host cell, of long-term survival under hostile extracellular conditions and of re-infecting a new host cell. By analogy to *E. coli*, it was proposed that *L. pneumophila*, when starved for amino acids, initiates a stringent response by synthesizing the second messenger guanosine tetraphosphate (p)ppGpp via the synthetase enzyme RelA (Hammer and Swanson, 1999). Indeed, a *L. pneumophila relA* mutant replicates efficiently within either amoebae or macrophages however upon entry into the post-exponential phase of growth, the mutant strain does not produce the second messenger (Zusman et al., 2002). Additionally, virulence traits are poorly expressed when *L. pneumophila* lacks RelA and consequently the alarmone (p)ppGpp (Hammer and Swanson, 1999; Zusman et al., 2002; Dalebroux et al., 2010a). The mild effects displayed by the lack of RelA on the expression of the virulent traits suggested that additional clues and redundant strategies are employed by *L. pneumophila* to govern its virulence (Hammer and Swanson, 1999; Zusman et al., 2002). Indeed *L. pneumophila* is equipped with two ppGpp synthetases, which respond to two distinct metabolic cues. Whereas, RelA synthesizes (p)ppGpp is following fluctuations in amino acid availability, the bifunctional enzyme SpoT leads to the accumulation of the alarmone (p)ppGpp in response to fatty acid depletion. By analogy to *E. coli*, a *L. pneumophila* strain depleted of *relA* and *spoT* lacks (p)ppGpp completely, however whether it results in rRNA transcriptional activation and/or in the synthesis of stable RNA remains unclear (Dalebroux et al., 2009; Dalebroux and Swanson, 2012; Trigui et al., 2015). Thus, the *L. pneumophila* biphasic life cycle requires the fine tuning of the levels of alarmone (p)ppGpp present in the bacteria. When nutrients are abundant, virulent bacteria hydrolyze (p)ppGpp in a SpoT-dependent manner, allowing the bacteria to actively multiply and repress the transmission traits (Molofsky and Swanson, 2003; Dalebroux et al., 2009, 2010a; Trigui et al., 2015). Conversely, as replicating bacteria exhaust the available nutrients within the LCV, (p)ppGpp is produced by RelA and additionally, the equilibrium of SpoT is shifted more toward synthesis instead degradation. This leads to a massive accumulation of the alarmone and triggers the entry into the transmissive state (Hammer and Swanson, 1999; Molofsky and Swanson, 2004; Dalebroux et al., 2009). SpoT is required throughout the entire infection cycle to mediate (p)ppGpp turnover *via* its hydrolase and synthase activities (Xiao et al., 1991; Potrykus and Cashel, 2008; Dalebroux et al., 2009, 2010a).

#### Transcriptional control by sigma factors

In *L. pneumophila* the signaling alarmone (p)ppGpp is a key player for the reorganization of the bacterial transcriptome by recruiting sigma factors, allowing the activation of genes necessary for the adaptation to the new condition and the repression of the ones that are no longer required (Dalebroux et al., 2010a). Particularly, the accumulation of (p)ppGpp increases the amount of the alternative sigma factor RpoS (σ^S/38^), which results in the regulation of multiple pathways associated with motility and pathogenic functions as well as the activity of transcriptional regulators and Dot/Icm effectors (Hales and Shuman, 1999; Bachman and Swanson, 2004; Trigui et al., 2015). The mechanism that links the accumulation of (p)ppGpp with the expression of RpoS remains to be elucidated, however (p)ppGpp is suggested to destabilize the binding of the vegetative sigma factor σ^D/70^ to the core and endorses the recruitment of alternative sigma factors and the expression of their targets, as demonstrated for *E.coli* (Jishage et al., 2002). An additional regulatory element, which acts as cofactor for (p)ppGpp-dependent transcriptional regulation is the RNA polymerase (RNAP) secondary channel interacting protein DksA (Haugen et al., 2008; Potrykus and Cashel, 2008). *L. pneumophila* DksA function may be dependent on bacterial stimuli. In particular, DksA seems to respond to fatty acid stress by inducing bacterial differentiation in a (p)ppGpp-independent manner, as judged by the expression of certain transmissive traits within macrophages (Dalebroux et al., 2010b). However, upon (p)ppGpp accumulation, DksA and (p)ppGpp coordinately regulate the hierarchical cascade for flagellar expression. Therefore, *L. pneumophila* employs both (p)ppGpp and DksA to act independently or cooperatively during bacterial differentiation (Dalebroux et al., 2010b). At the bottom of the hierarchical cascade governing *L. pneumophila* differentiation one can find the flagellar regulon, composed of four different classes of genes, whose coordinated expression is crucial for efficient and maximal virulence of the bacterium (Heuner et al., 1997; Dietrich et al., 2001; Brüggemann et al., 2006; Appelt and Heuner, 2017). Class I genes, which include the genes encoding the flagellar master regulator and the σ^54^ activator protein FleQ together with the alternative sigma factor RpoN (σ^54^), are required for the expression of the class II genes, leading to the formation of the flagellar basal body, hook and the activation of the regulatory proteins (Jacobi et al., 2004; Steinert et al., 2007; Albert-Weissenberger et al., 2010). Finally, the flagellar sigma factor FliA (σ^28^) (encoded by a class III gene and regulated by DksA) is directly controlling the flagellar class IV genes such as *flaA* and *fliDS*, encoding the flagellin and the filament cap respectively, leading to the complete formation of the flagellum (Heuner and Steinert, 2003; Jacobi et al., 2004; Brüggemann et al., 2006; Albert-Weissenberger et al., 2007, 2010; Dalebroux et al., 2010b). Interestingly, the flagellar sigma factor FliA is not only implicated in the regulation of the flagellum production but also acts as regulator of virulence genes that are required for the expression of pathways important for cytotoxicity, lysosome evasion, and replication of *L. pneumophila* (Heuner et al., 2002; Molofsky and Swanson, 2004; Brüggemann et al., 2006).

#### Post-transcriptional regulation of transmissive traits

**Figure 3:**
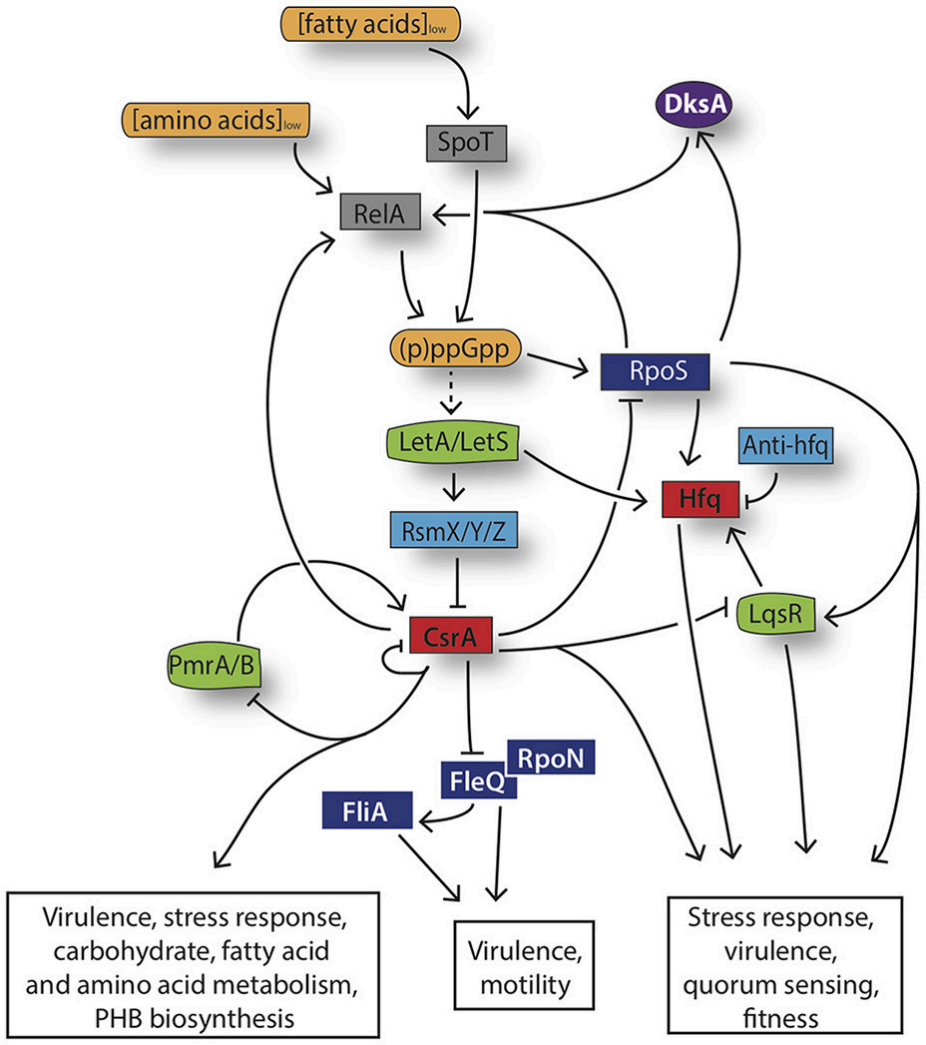
Model the stringent response network governing *L. pneumophila* differentiation. Amino acid and fatty acid starvation triggers RelA and SpoT to produce the alarmone (p)ppGpp. Its accumulation induces the activation of the stress sigma factor RpoS, the LetA/LetS TCS and consequently an increased transcription of the RsmZ, RsmX, and RsmY sRNAs. The three sRNAs act as “sponge,” sequestering CsrA and leading thereby to the activation of the infectious traits and to changes in the metabolism. The dashed arrows indicate suggested but not yet confirmed direct interactions.

As in many other bacterial pathogens, *L. pneumophila* post-transcriptional regulation is controlled by two-component systems (TCS), which use protein phosphorylation cascades for signal transduction (Padilla-Vaca et al., 2017). *L. pneumophila* employs at least four distinct TCSs LetA/S, PmrA/B, LsqR/ST, and CpxR/A that govern its differentiation from the replicative to the transmissive state (Gal-Mor and Segal, 2003a; Tiaden et al., 2007; Zusman et al., 2007; Altman and Segal, 2008). Particularly, the TCS LetA/LetS (*Legionella* transmission activator and sensor, respectively) of *L. pneumophila* is an important regulator system for the activation of a large set of virulence phenotypes and the control of the progression into the transmissive state (Hammer et al., 2002; Gal-Mor and Segal, 2003b; Lynch et al., 2003). Probably directly activated by the accumulation of the alarmone (p)ppGpp, LetA is regulating the expression of the small ncRNAs RmsX,Y,Z, which are required to relieve the repression exerted by the global regulator CsrA, an RNA-binding protein, on many virulence genes, thereby ensuring the expression of the transmissive traits (Hovel-Miner et al., 2009; Rasis and Segal, 2009; Sahr et al., 2009; Edwards et al., 2010). The carbon storage regulator protein CsrA was reported to bind more than 450 mRNA targets in *L. pneumophila*, altering their translation, transcription and/or their stability (Sahr et al., 2009, 2017). Among those targets, CsrA affects the expression of the previously mentioned regulators FleQ, RpoS, the quorum sensing regulator LqsR and it also control the expression of over 40 Dot/Icm substrates (Sahr et al., 2017). Moreover, CsrA controls its own expression and the *relA* mRNA in a regulatory feedback loop. This in turn makes the CsrA protein indispensable for *L. pneumophila* thus only conditional or partial mutants could be obtained, which are all however strongly attenuated for intracellular multiplication, underlining its essential role in the life cycle of *L. pneumophila* (Molofsky and Swanson, 2003; Sahr et al., 2017). Another TCS important for virulence gene expression is PmrA/B (Zusman et al., 2007). *L. pneumophila* PmrA/B not only activates the expression of 43 effector-encoding genes but also positively regulates CsrA and consequently post-transcriptional repression of the CsrA- regulated effectors (Zusman et al., 2007; Al-Khodor et al., 2009; Rasis and Segal, 2009) (Figure 3). It is likely that a regulatory switch between at least two sets of effectors occurs: one set of effectors, activated by PmrA/B and expressed in the replicative state and the second group of effectors which is regulated by the LetA/S TCS upon entry into the transmissive phase of *L. pneumophila*. Another player in this complex regulatory network, is the TCS LqsRS (*Legionella* quorum sensing), whose role in the regulation of gene expression during the transmissive phase has been extensively studied (Hochstrasser and Hilbi, 2017). Importantly, the production of LqsR is regulated at the post-transcriptional level by the global repressor CsrA (Sahr et al., 2009, 2017). Finally, the CpxR/A TCS, which acts as dual regulator and thus as an activator and repressor, was shown to control the expression of at least 27 Dot/Icm substrates as well as type II- secreted virulence factors, playing a important role in *L. pneumophila* virulence gene regulation (Gal-Mor and Segal, 2003a; Altman and Segal, 2008).

In addition to TCSs and the RNA-binding protein CsrA, another major player in the regulation of the transition from replicative to transmissive *L. pneumophila* is the RNA binding protein and chaperone Hfq (McNealy et al., 2005; Trigui et al., 2013). This pleiotropic regulatory element is known to modulate gene expression by facilitating the interaction between sRNA and their mRNA targets in diverse bacterial pathogens, controlling pathways related to metabolism, transport, energy production and conversion or membrane proteins (Boudry et al., 2014). In *L. pneumophila*, Hfq expression is influenced by RpoS and LetA regulatory elements as both directly or indirectly turn on *hfq* transcription upon onset of the late post-exponential phase. Furthermore, *L. pneumophila* Hfq regulates its own expression in an auto-regulatory loop (Oliva et al., 2017). Although, only two direct targets *(hfq* mRNA and Anti-hfq sRNA) of *L. pneumophila* Hfq have been identified to date by *in vitro* assays, Hfq was reported to regulate the bacterium's virulence, as judged by the findings that this post-transcriptional regulator promotes motility and is required for efficient multiplication of *L. pneumophila* within *A. castellanii* at environmental temperatures (McNealy et al., 2005; Oliva et al., 2017) (Figure 3).

#### *L. pneumophila* engages sRNAs to control its virulence

The complex and hierarchical regulation of the *L. pneumophila* life cycle includes also the recruitment of small RNAs, which ensure a fast and more cost-effective regulation. Previous evidences in *E. coli* showed that the BarA/UvrY TCS (the *L. pneumophila* LetA/S homolog) controls the expression of two sRNAs, named CsrB and CsrC, whose sequences contain GGA motifs, which are the characteristic binding sequences for CsrA (Liu et al., 1997). A first bioinformatics search revealed in *L. pneumophila* the presence of two homologs of CsrB, named RsmY and RsmZ (Kulkarni et al., 2006). Functional analyses confirmed that these sRNAs were the missing regulatory elements linking the LetA/S TCS and the RNA binding protein CsrA in *L. pneumophila* (Rasis and Segal, 2009; Sahr et al., 2009). In detail, LetA binds directly to a conserved consensus sequence upstream the *rsmY/Z* genes, leading to their expression. These sRNAs contain multiple CsrA binding motifs and act as sponge to bind and sequester CsrA from their target mRNAs, leading to the expression of virulence traits. RsmY and RsmZ were the first characterized sRNAs implicated in the regulation of *L. pneumophila* virulence. However, deep RNA sequencing from exponentially (replicative) and post exponentially (virulent) *in vitro* grown *L. pneumophila* have identified more than 700 sRNAs, 60% of which are growth-phase dependently regulated, including a third LetA-dependent sRNA, named RsmX, suggesting that a set of these yet uncharacterized sRNAs, might influence the expression of replication or virulence determinants in *L. pneumophila* (Sahr et al., 2012). Recently, we characterized one of these ncRNAs, a *cis*-encoded sRNA for which we showed that it is implicated in the regulation of the RNA binding protein Hfq (Oliva et al., 2017). This sRNA, named Anti-hfq, is transcribed antisense to the *hfq* transcript and controls the expression of Hfq through a base pairing mechanism during the exponential phase of *L. pneumophila* growth (Oliva et al., 2017). Moreover, it is important to mention that Hfq was reported to influence *L. pneumophila* differentiation by interacting with the major regulatory elements of the cascade. Thus, it is expected that Hfq, acting as RNA chaperone and RNA binder might regulate a number of still unknown sRNAs implicated in bacterial virulence.

Taken together, *L. pneumophila* is equipped with a sophisticated regulatory network, including transcriptional and post-transcriptional regulatory elements, including small non-coding sense and antisense RNAs to control the reciprocal expression of distinct sets of genes under different environmental conditions (Figure 3).

## Cross talk between metabolism and the stringent response

Similarly to what has been described in other bacterial pathogens, many regulatory factors implicated in virulence gene expression are also major regulators of metabolic pathways. Indeed, *L. pneumophila* exhibits a bipartite metabolism, which requires a fine-tuned regulation. An intriguing example of a regulator that is important for the expression of virulence and the regulation of metabolic traits is the RNA binding protein CsrA. Interestingly, *L. pneumophila* harbors some of the key genes encoding enzymes of the glycolysis/gluconeogenesis (glyceraldeyde-3-phosphate dehydrogenase or Gap, phosphoglycerate kinase, and pyruvate kinase) and the PPP (transketolase) in one single operon. The combined or individual regulation of these two pathways is under the control of the RNA binding protein CsrA, whose presence ensures the efficient expression of the both parts of this operon (Sahr et al., 2017). When nutrients are abundant CsrA binds within the *gap* transcript, and stabilizes the alternative secondary structure that covers the Rho-dependent transcription termination site. Consequently, this leads to a CsrA-dependent transcription of the glycolysis part of the operon toward gluconeogenesis, which under starvation or stress is not expressed. Another example of how CsrA influences metabolism, is that this regulatory element affects the production of secondary metabolites, in particular thiamine pyrophosphate, ensuring the effecting functioning of central enzymes of the carbohydrate metabolism when required (Sahr et al., 2017).

Indeed, using ^13^C-isotopologue profiling and carbon-flux analyses of a wild-type and a *csrA* mutant strain confirmed that CsrA plays a major role in regulating the carbon flux between the PPP and the glycolysis (Häuslein et al., 2017). Furthermore, this study highlighted the impact of CsrA on the bipartite metabolism of *L. pneumophila*, as the absence of CsrA induces a reduction of the carbon flux from serine *via* gluconeogenesis into the PPP. By contrast, CsrA has a negative impact on the incorporation and the metabolism of glycerol and glucose. As such, the absence of CsrA results in the increase of the carbon flux from glucose into the PPP and ED pathways and the carbon flux from glycerol into the PPP and the gluconeogenesis (Häuslein et al., 2017). These studies also showed the important influence of CsrA on the production of the storage molecule PHB suggesting that CsrA is a major player in the utilization of the different carbon sources during the biphasic life cycle of *L. pneumophila* (Häuslein et al., 2017). The biphasic life cycle of *L. pneumophila* within the host supports the usage of amino acids as main carbon and energy source during multiplication due to the expression of CsrA that is simultaneously repressing the usage of alternative carbon sources, such as glycerol. Conversely, upon onset of the post-exponential phase of growth, the stress response induces the sRNA RsmX, Y, and Z that sequester CsrA, resulting in an increased utilization of glycerolipids, which along with glucose, mostly trigger the synthesis of lipopolysaccharide sugars through the PPP and in addition, the production of the energy and carbon storage polymer PHB (Häuslein et al., 2017). Hence, CsrA is a major organizer of the biphasic life cycle of *Legionella pneumophila* integrating and coordinating the metabolic carbon switch and the transition between replicative and transmissive traits.

## Concluding remarks

*L. pneumophila* is an intracellular opportunistic pathogen, which exploits amoebae and other protozoa as environmental hosts, but that is also able to infect human macrophages, eventually causing Legionnaires' disease, a severe pneumonia that is often fatal when not treated promptly. *L. pneumophila* is ubiquitously found in fresh water habitats, as planktonic form or forming biofilm. In response to diverse and hostile environmental conditions encountered during its life cycle, *L. pneumophila* has evolved sophisticated mechanisms to successfully replicate within different host niches and to also survive in extracellular environments. As such, this intracellular bacterium displays at least two reciprocal stages: a replicative and a transmissive form. The transition between the non-virulent replicative and the virulent non-replicative phase is governed by a complex regulatory network, in which transcriptional and post-transcriptional regulatory elements are engaged to insure an efficient infection cycle. The trigger of this morphological stress response is mainly mediated by metabolic changes and therefore the availability of nutrients in the surroundings. Thus, within the LCV the usage of serine as carbon and energy source supports the multiplication of the bacteria in which the replicating bacteria show a high metabolic activity. Upon amino acid depletion, the stringent response mediates the expression of the virulent traits but in parallel also enables the bacteria to survive for long term under stress and starving condition. This is ensured amongst others by the expression of stress and virulence related genes and an overall metabolic shift leading to the usage of alternative carbon sources like glucose and glycerolipids and an increased production of the storage molecule PHB. Under these conditions, *L. pneumophila* is optimally equipped to escape the spent host, survive for an uncertain period in the extracellular environment and eventually start a new infection cycle.

Taken together, the biphasic life cycle of *L. pneumophila* results in distinct morphological changes and a bipartite carbon metabolism. Thus, during the biphasic life cycle the metabolism influences the transition between replicative and transmissive phase as well as the reciprocal expression of virulence factors and their regulators, in particular CsrA, which is implicated in the regulation of virulence and the metabolism. A comprehensive analysis of *L. pneumophila* adaptation to metabolic cues during the transmissive phase *in vivo* either in amoebae or macrophages is still missing and would provide additional information about the utilization of diverse carbohydrates, and the cross-talk of the regulatory elements which govern *L. pneumophila* virulence. Continuous unrevealing of this complex interplay between metabolism and virulence of *L. pneumophila* may teach us also about host-pathogen interaction in general.

## Author contributions

All authors listed have made a substantial, direct and intellectual contribution to the work, and approved it for publication.

### Conflict of interest statement

The authors declare that the research was conducted in the absence of any commercial or financial relationships that could be construed as a potential conflict of interest.
